# Characterizing Normal and Pathological Gait through Permutation Entropy

**DOI:** 10.3390/e20010077

**Published:** 2018-01-19

**Authors:** Massimiliano Zanin, David Gómez-Andrés, Irene Pulido-Valdeolivas, Juan Andrés Martín-Gonzalo, Javier López-López, Samuel Ignacio Pascual-Pascual, Estrella Rausell

**Affiliations:** 1 Center for Biomedical Technology, Universidad Politécnica de Madrid, Pozuelo de Alarcón, 28223 Madrid, Spain; 2 Department of Computer Science, Faculty of Science and Technology, Universidade Nova de Lisboa, 2829-516 Lisboa, Portugal; 3 MOVUAM-TRADESMA laboratory, Department of Anatomy, Histology and Neuroscience, Universidad Autónoma de Madrid, IdiPaz, 28029 Madrid, Spain; 4 Paediatric Neurology Research Group, Hospital Universitari Vall d’Hebron, VHIR, 08035 Barcelona, Spain; 5 Center of Neuroimmunology and Department of Neurology, Hospital Clínic of Barcelona, Institut d’Investigacions Biomèdiques August Pi Sunyer (IDIBAPS), Universitat de Barcelona, 08036 Barcelona, Spain; 6 Escuela Universitaria de Fisioterapia de la ONCE-UAM, 28034 Madrid, Spain; 7 Department of Physical Medicine and Rehabilitation, Hospital Universitario Infanta Sofía, San Sebastián de los Reyes, 28702 Madrid, Spain; 8 Servicio de Neuropediatría, Hospital Universitario La Paz, 28034 Madrid, Spain

**Keywords:** permutation entropy, cerebral palsy, instrumental gait analysis

## Abstract

Cerebral palsy is a physical impairment stemming from a brain lesion at perinatal time, most of the time resulting in gait abnormalities: the first cause of severe disability in childhood. Gait study, and instrumental gait analysis in particular, has been receiving increasing attention in the last few years, for being the complex result of the interactions between different brain motor areas and thus a proxy in the understanding of the underlying neural dynamics. Yet, and in spite of its importance, little is still known about how the brain adapts to cerebral palsy and to its impaired gait and, consequently, about the best strategies for mitigating the disability. In this contribution, we present the hitherto first analysis of joint kinematics data using permutation entropy, comparing cerebral palsy children with a set of matched control subjects. We find a significant increase in the permutation entropy for the former group, thus indicating a more complex and erratic neural control of joints and a non-trivial relationship between the permutation entropy and the gait speed. We further show how this information theory measure can be used to train a data mining model able to forecast the child’s condition. We finally discuss the relevance of these results in clinical applications and specifically in the design of personalized medicine interventions.

## 1. Introduction

Cerebral Palsy (CP), the most frequent cause of severe disability in childhood, is the result of a single brain lesion at perinatal time, which may affect the brain to various degrees [1]. Subsequent movement impairments, in particular gait abnormalities, are very frequent, altering daily life and causing dependency [2]. Joint movements during gait are tightly controlled by neural motor brain structures. As a whole, these brain structures regulate many factors contributing to the appropriate sequence of flexor-extensor muscle contractions, the final result being a precise movement of the joint chains and the translation of the body’s center of gravity in bipedal posture [3]. Patients with CP have suffered a brain injury during early development in one or several cerebral areas that are important for motor control. The most common is the corticospinal tract, formed by axons of the cerebral cortex motor neurons. This pathway travels all the way to the spinal cord and regulates the output signals of the postsynaptic motor neuronal circuitry and, therefore, the sequence of muscle contraction [4]. Gait motor performance is hence altered at first instance, impeding the correct translation of the body. However, gait is a cognitive propositive action of high importance for the brain, and the whole nervous system adapts its dynamics to achieve the target. Several plastic mechanisms are then triggered, including, for instance, the re-arrangement of synaptic connections [4]. This ultimately results in the generation of newly-configured sequences of muscle contractions, executing a sort of “maladaptive” gait. Therefore, while the cognitive target is achieved, the movement might not be properly adjusted to the biomechanical properties of the joint’s soft tissues, muscles and bones and to cellular metabolic needs, thus yielding torque-related deformities. Those newly-generated signals are a reflection of brain adaptation, and the quantitative evaluation of the differences between the CP patients’ and the control subjects’ signals is essential for the interpretation of the prognosis of the disease and for the design of personalized therapies.

These considerations have led to the development of Instrumental Gait Analysis (IGA), a set of techniques that are able to objectively quantify human gait. Its use is gaining followers in clinical practice and research. IGA assesses patient’s specific problems by measuring how the body moves as a whole and providing spatio-temporal parameters (e.g., walking speed or step length); and by further acquiring high frequency kinematic measurements of those joints that align the lower extremity segments along the patient’s gait cycle: the period of time between two consecutive floor-heel contacts of the same foot [5]. More importantly, IGA results, in terms of gait cycles, are sets of time series that can quantitatively and objectively be studied using the large repertoire of algorithms offered by information theory.

As for any signal-dependent biological system, joint movements during gait are the sum of stochastic and deterministic influences [6]. It thus seems only natural to resort to entropy measures for the analysis of IGA signals, as they are tailored to the characterization of the presence and balance of those influences. Entropy measures can be useful to quantify the performance of the motor system’s signal in selecting joint motions along the gait cycle: a key dimension for motor control [7,8]. A joint movement with low entropy is a more repeatable, less erratic one, suggesting that it is tightly controlled by its corresponding neural command, while at the same time it is less adaptable or plastic to internal or external modifiers [9]. Along these lines, entropy analysis has previously been applied to IGA data obtained from CP patients, with the aim of, e.g., assessing the repeatability of signals such as superficial Electromyography (EMG) synchronized with gait analysis [10,11], which describes the activation of the different muscles by the central nervous system. EMG signals are difficult to acquire consistently, and therefore interpreted, since many factors influence their quality, like the type of electrodes and their exact position on the muscle along the nerve path. There are nevertheless several other signals that are relevant to understanding the nature of brain adaptations in CP patients. Specifically, joint kinematic data, which are easy to acquire and reflect the level of movement complexity induced by the neural signal, have received little attention so far.

In this paper, we present the first (to the best of our knowledge) analysis of the joint kinematics during the gait cycle of healthy children and children with bilateral spastic CP, through the assessment of the signal’s single-scale and multi-scale Permutation Entropies (PEs) [12,13]. As opposed to past research studies, in which similar data were analyzed through other entropy measures [14,15,16], PE has here been chosen for its capacity for characterizing the temporal dynamics of the system, a key aspect of movement assessment [17], without the need for arbitrary thresholds or binning.

Our aim is to study PE as a measurement of motor control, thus being complementary to existing indexes for the assessment of motor impairment. We used traditional statistical methods and diagnostic models based on data mining algorithms to demonstrate a significant increase in the permutation entropy of CP children’s movements. The magnitude of such an increase depends in a non-trivial way on the joint analyzed and varies across the spectrum of functional disability, suggesting the existence of different adaptive mechanisms in the lesioned motor system. In addition, we found that the permutation entropy is negatively correlated with the walking speed, in both groups of children. CP children walk slower than healthy children, so that their entropy values are higher, but we demonstrate that having a CP condition increases this effect in some joint gait movements. We believe that the use of PE opens new doors towards the analysis of motor control in healthy subjects and patients, with potential clinical and therapeutic implications.

## 2. Results

### 2.1. Gait Permutation Entropy: Single-Scale

We start with an analysis of the differences between our control subjects and CP patients in terms of gait single-scale permutation entropy for each movement; see Section 4.1 and Section 4.2.1 for a description of the data and of the methodology. In brief, we calculated single-scale permutation entropy of the time series (gait cycles) of five joints (pelvis, hip, knee, ankle and forefoot) in three planes of movement (abduction-adduction in the coronal plane, flexion-extension in the sagittal plane and medial-lateral rotation in the horizontal plane) during the gait cycle (between two consecutive heel contacts). We acquired 229 cycles from 27 healthy children (our published database of normality) and 455 cycles from 53 CP children recruited in our reference hospitals (GMFCS I: 5 patients, GMFCS II: 19 patients, GMFCS III: 23 patients and GMFCS IV: 6 patients). Note that GMFCS refers to the Gross Motor Function Classification System, a standard scale for assessing movement disability [18]. Permutation entropy was independently calculated in each cycle using 201 values of the corresponding angular joint positions. We have described the distribution and compared the differences of PE between healthy and CP children. Moreover, we have studied the relationships of PE with CP children’s kinematic abnormality and degree of functional dependence, the latter being encoded by the GMFCS stage.

**Figure 1:**
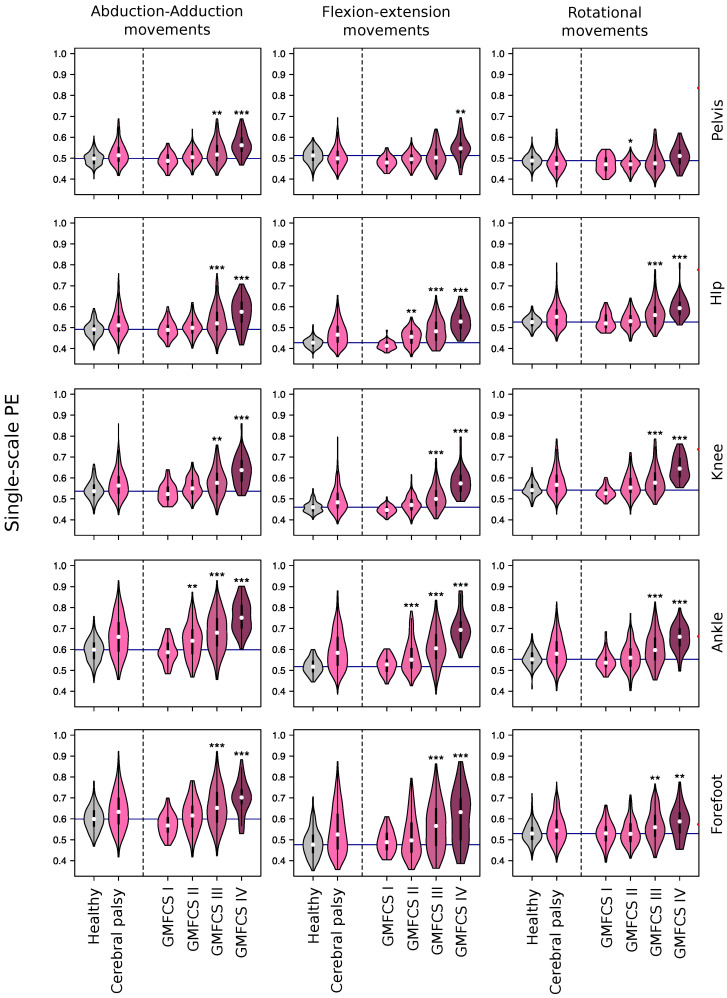
Probability distributions of the Permutation Entropy (PE), as calculated in control subjects and Cerebral Palsy (CP) patients, the latter including aggregated and disaggregated (w.r.t. the Gross Motor Function Classification System (GMFCS) scale) results. Rows, from top to bottom, respectively correspond to pelvis, hip, knee, ankle and forefoot; columns, from left to right, to the abduction-adduction, sagittal and rotational axes.

Figure 1 reports 15 violin plots [19], each one describing the probability distributions of the computed PE values as measured in control subjects and patients. Rows correspond to the five considered joints (pelvis, hip, knee, ankle and forefoot) and columns to the three axes (abduction-adduction, sagittal and rotational). The two left-most violin graphs of each panel correspond to control subjects and patients, the latter being aggregated irrespective of the CP severity; this latter distribution is then disaggregated in the four remaining violin plots, according to the output of a GMFCS assessment.

In healthy subjects, single-scale PE varies according to the movement plane. Adduction-abduction and rotational movements tend to have greater values of PE than flexion-extension movements. The same applies to distal joints (ankle and forefoot) in comparison to proximal joints (knee and hip).

**Figure 2:**
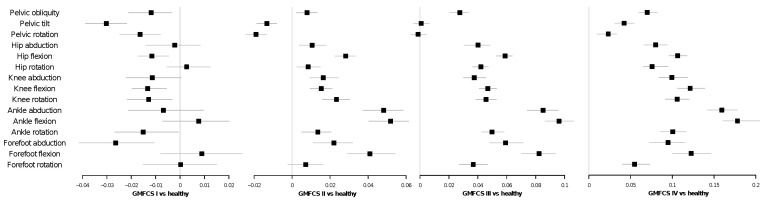
Forest plots showing the beta coefficients of linear mixed models comparing gait PE values according to the patient’s GMFCS level. Squares represent the mean value of each beta coefficient and horizontal lines the corresponding 95% bias-corrected and accelerated bootstrap intervals. See main text and Section 4.3 for details.

In general, the permutation entropy of patients, averaged over their corresponding cycles, is higher than that of control subjects. This difference is further confirmed by Table 1, reporting the *p*-values of two-sided *t*-tests for the null hypothesis that patients and control subjects have an identical PE average. The four disaggregated violin plots also suggest that the PE is correlated with the GMFCS scale, with Levels III and IV (i.e., children walking using a hand-held mobility device or requiring physical assistance) showing the highest entropy values. This is further confirmed in Figure 2, which includes forest plots depicting the results of linear mixed models comparing gait PE values according to the patient’s GMFCS level. Beta scores, when higher than zero, indicate an increase in the PE mean of each GMFCS level w.r.t. the control group; see Section 4.3 for details. In agreement with Figure 1, all betas for the GMFCS IV level are positive and statistically significant. PE produces a measure of complexity (or unpredictability) of a time series. Thus, Figure 1 and Figure 2 indicate that patients display more complex, possibly less controlled and more erratic movements.

**Figure 3:**
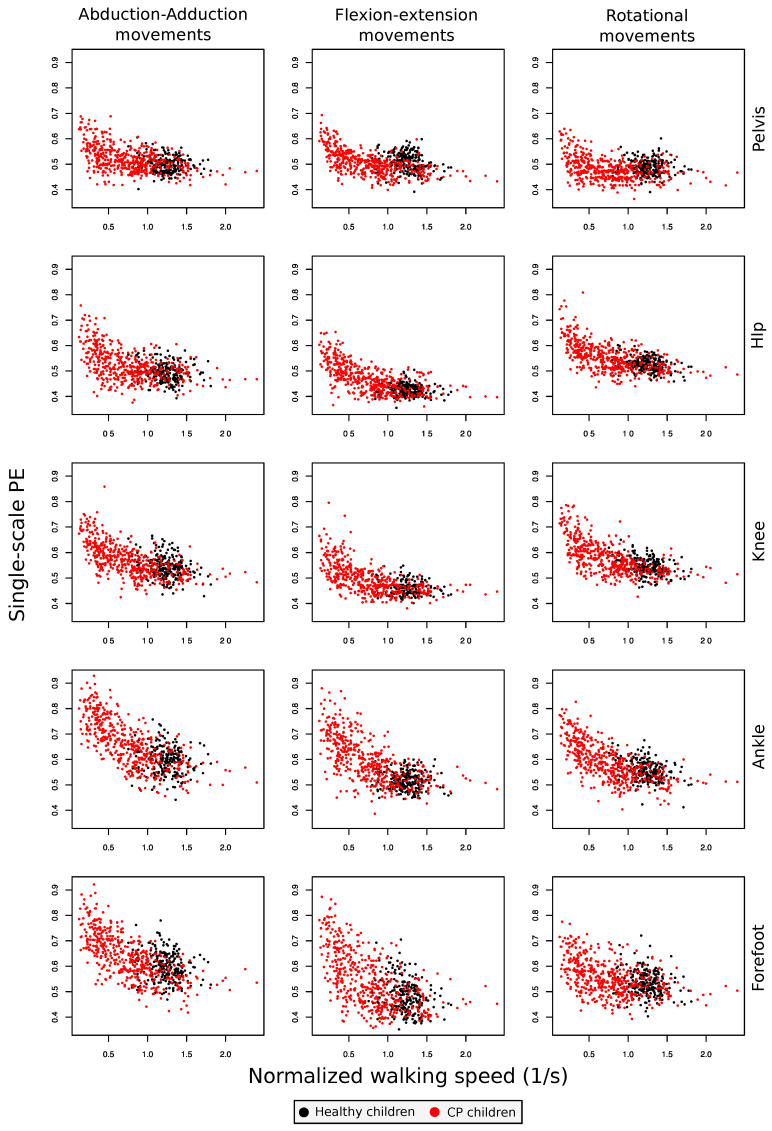
Permutation entropy as a function of the normalized walking speed, for control subjects (black dots) and CP patients (red dots). Each panel corresponds to the same joint/axis as in Figure 1.

**Figure 4:**
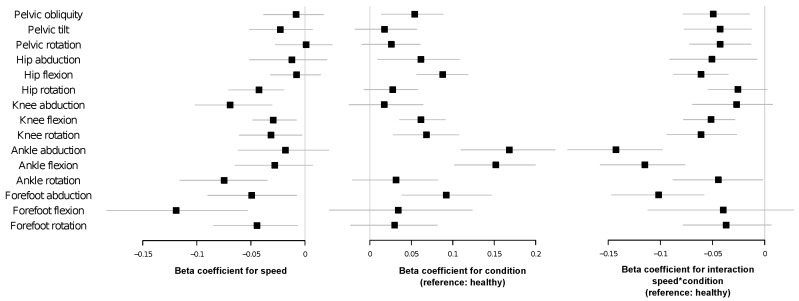
Forest plots showing the beta coefficients of linear mixed models comparing gait PE values according to normalized walking speed (left panel), condition (using healthy as the reference, central panel) and the interaction of normalized walking speed with condition (right panel). The magnitudes of the effects are indicated on the X axis. Squares represent the mean values of each beta coefficient and horizontal lines the corresponding 95% bias-corrected and accelerated bootstrap intervals. See Section 4.3 for further details.

In order to better understand the relationship between the observed PE and other gait characteristics, Figure 3 presents a series of scatter plots depicting the entropy of each gait cycle as a function of its normalized walking speed (walking speed/lower limb length). Each panel corresponds to the same joint/axis as in Figure 1; and black and red points respectively correspond to control subjects and patients. A clear linear/quadratic relationship can be observed, with high PE values usually associated with low walking speeds, which are generally preferred by CP patients. We investigated this relationship more carefully with a set of linear mixed models. These models describe the effect of the normalized walking speed on PE, the effect of the condition of having CP and the effect of interaction of normalized walking speed with condition; see Figure 4 for results, and Section 4.3 for details on the methodology. As the impact of each one of these three variables is modeled independently of the others, this methodology allows one to estimate whether the PE values we observe are just due to a reduced gait speed (a characteristic of CP patients) or are further explained by the presence of the condition. As indicated by the negative beta scores for speed in these models (left panel of Figure 4), PE seems to be negatively correlated with speed. This negative correlation is significant for hip rotation, knee adduction, knee flexion, knee rotation, ankle rotation and all movements of the forefoot. The effect of suffering from CP is shown both in beta scores for condition (central panel of Figure 4) and in beta scores for interaction (condition multiplied by speed) (right panel of Figure 4). With the exception of hip abduction, knee abduction, forefoot flexion and rotation, PE values are significantly different in patients with CP. On the other hand, in the case of these exceptions, suffering from CP does not significantly alter the average PE value (beta score for the condition is not significant), but it makes the impact of speed on PE even more intense (beta score for interaction is significant). In the specific case of pelvic tilt and rotation, the increase in PE is due to a combination of slower walking speed and condition, while the two same elements, when considered separately, are not significant per se. In other cases (i.e., knee flexion), both beta scores (condition and interaction) are significant. This means that the rate of increase in PE with slower walking speed is higher than in healthy subjects and that for speed near zero, PE values would be expected to be higher in CP.

There are several possible explanations for these relationships of PE with walking speed, which are not mutually exclusive. Firstly, normalized walking speed is merely related to PE because both speed and PE are related to the severity of motor alteration in CP. This is supported by the relationship of PE with GMFCS. Secondly, speed has an influence on PE, and those subjects who cannot walk faster due to the motor impairment recruit neural gait strategies with lower PE values. Thirdly, CP patients recruit gait strategies that have higher PE and slow preferred walking speed without any direct relationships between these two factors.

A complete explanation would require a full causality analysis and possibly a specific experiment in which the gait speed is forced to be constant, which would entail several technical and medical difficulties and is beyond the scope of this work. Nevertheless, the results presented in this section allow us to confidently conclude that, above and beyond the difference in speed, patients’ gait is less predictable (and less controlled) than that of control subjects; additionally, such lack of predictability seems to convey information about the progression of the disease not readily available in the gait speed.

**Figure 5:**
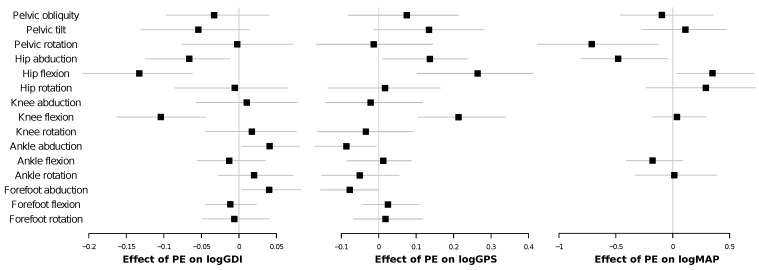
Forest plots showing the beta coefficients of linear mixed models that measure the effect of PE on the Gait Deviation Index (GDI, left), the Global Profile Score (GPS, center) and elements of the Movement Analysis Profile (MAP, right). The magnitudes of the effects are indicated in the X axis. Squares represent the mean values of each beta coefficient and horizontal lines the corresponding 95% bias-corrected and accelerated bootstrap intervals. See Section 4.3 for further details.

We also explored the relationships between single-scale PE and gait indices in children with CP (see Figure 5). Gait indices are metrics that indicate how far the kinematic performance of one patient’s cycle is from a normal dataset used as the reference. We studied the relationships between PE and two indices of global gait kinematic performance, the Gait Deviation Index (GDI) and the Global Profile Score (GPS). The lower the GDI value, or the greater the GPS value, the more abnormal the shape of the kinematic curves is. We found that increased values of PE in hip abduction, hip flexion and knee flexion and lower values of PE in ankle and forefoot abduction-adduction are related to increased global gait kinematic impairment. Additionally, we studied the relationship of each gait cycle PE value with the corresponding item of the Movement Analysis Profile (MAP), which assesses the distance from normalcy of nine particular joint time series instead of analyzing the global performance, as GDI or GPS do. Our results suggest that PE and movement kinematic abnormality describe different and complementary aspects of the CP condition and that they are not always correlated. The whole relationship spectrum includes, on one end, the case of pelvic rotation and hip abduction, for which an increase in the movement abnormality leads to less erratic movement, as if that maladaptive movement were recruited to increase accuracy during gait. On the other end, only for hip flexion, there is a significant correlation between the abnormality of the joint movement and the PE.

### 2.2. Gait Permutation Entropy: Multi-Scale

Although the standard permutation entropy, as defined in Section 4.2.1, is an excellent tool to understand the complexity and global dynamics of a system, it yields little information regarding the time scale of such dynamics. We here therefore complement the previously obtained results with a multi-scale analysis, in which the time series are down-sampled according to different time resolutions υ, for then calculating the corresponding multi-scale PE; see Section 4.2.2 for details.

**Figure 6:**
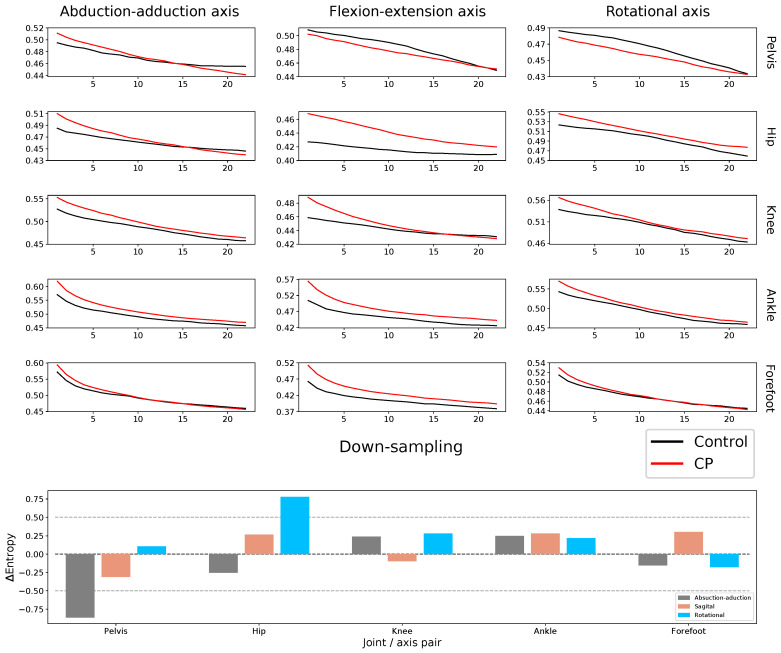
(Top) Multi-scale PE, for control subjects (black lines) and CP patients (red lines), as a function of the down-sampling υ; see Section 4.2.2 for details. Each panel corresponds to the same joint/axis as in Figure 1. (Bottom) ΔMSE for all joint/axis pairs; see Equation (1).

The top panels of Figure 6 depict the progression of the PE, as a function of υ, for control subjects and patients; each panel represents the same joint/axis as in Figure 1 and Figure 3. Overall, the PE values tend to decrease with υ for both healthy and CP individuals; this is to be expected, as the down-sampling process deletes information about high frequencies and creates smoother time series. Additionally, the entropy drop in CP patients is always greater than in control subjects. To confirm this point, the bottom graph depicts the drop in multi-scale entropy, ΔMSE, a metric defined for each joint/axis pair as:

(1) $$\Delta \mathrm{MSE}=\frac{MSE_{CP}\left(\upsilon =22\right)-MSE_{C}\left(\upsilon =22\right)}{MSE_{CP}\left(\upsilon =1\right)-MSE_{C}\left(\upsilon =1\right)},$$

$MSE_{C}\left(\upsilon =i\right)$ and $MSE_{CP}\left(\upsilon =i\right)$ respectively are the multi-scale PE of control subjects and CP patients for υ equal to *i*. As the purpose of this metric is to describe how the entropy evolves at different time scales, the specific value of υ=22 is not relevant per se, and similar results have been obtained for 22>υ>1. It is however important to note that, the higher υ, the shorter the resulting time series; as a downsampling process is performed. The value of υ=22 has thus been chosen to be the largest one ensuring the statistical significance of the results; as time series with a length of at least (D+1)!=4!=24 are required [21].

Values of ΔMSE close to zero, as indeed found in most cases, indicate that the initial difference between control subjects and patients, i.e., as yielded by MSE(υ=1), is reduced by the down-sampling process (with υ=22). In the latter case, i.e., for large υs, the down-sampling process results in an attenuation of the high-frequency components of the signal: therefore, while the overall envelope of the joint movement (or its macroscopic shape) is similar, patients suffer from a lack of high frequency and high precision control.

### 2.3. Gait Entropy in Classification Tasks

If the previous analyses have shown that there is a (statistically significant) difference between control subjects and CP patients in terms of gait PE, it is less clear whether this difference has just a descriptive value or can also be used in predictive tasks. The difference between the two aspects is far from trivial, as has been shown in recent works; the interested reader may refer to Section 3.1 of [22] for further discussions. We here clarify this point by assessing whether a classification model can be constructed, using the PE values as input features and able to correctly classify new individuals according to their health condition.

**Figure 7:**
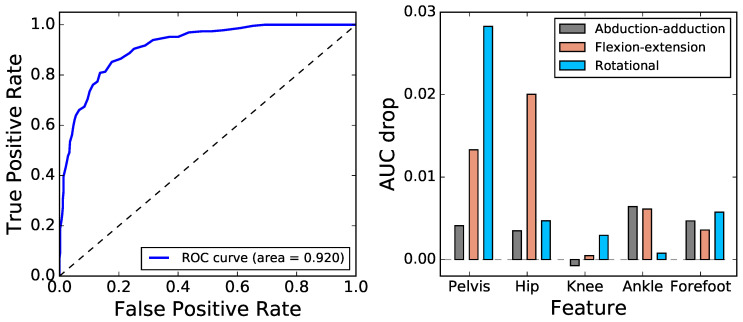
Classifying patients according to their gait entropy. The left panel depicts the Receiver Operating Characteristic (ROC) curve (blue solid line), obtained through a random forest model; the dashed grey line represents the result obtained by a random classification. The right panel depicts the drop in the Area Under the Curve (AUC) when individual features (joint/axis pairs) are deleted from the dataset; the higher the value, the more important is the considered feature.

As a first approximation, each individual *i* is here represented by a vector $f_{i}$, of size 15, encoding his/her corresponding single-scale PE; and by a scalar $c_{i}$ defining his/her class (i.e., $c_{i}=0$ when the person is a control subject, and $c_{i}=1$ when he/she suffers from CP). This information is used to train a Random Forest (RF) classifier (see Section 4.4), which has been validated through a leave-one-out cross-validation strategy. Figure 7 (left, blue line) depicts the Receiver Operating Characteristic (ROC) curve, a standard way of representing the relationship between sensitivity and fall-out of a binary classifier. The area under this curve (Area Under the Curve, AUC =0.920) is well above that expected in a random classification (i.e., 0.5) and thus indicates that the single-scale PE could differentiate between healthy and CP children.

Figure 7 (right) further depicts the drop in the AUC, when one of the 15 considered features is deleted from the training data; in other words, this value represents how essential (or important) a given feature is in the classification. It can be perceived that the highest values are associated with the flexion-extension and rotational axes, especially for pelvis and hip. While these results generally correlate well with those of Figure 1, some exceptions can be found; as for instance for the abduction-adduction axis of the ankle, where the difference between control subjects and patients is strong in Figure 1, but the relevance of which in the classification is small. Such discrepancies are due to redundancies in the data: other features can explain the entropy of the one considered, and therefore, its deletion is not accompanied by a reduction in the classifier’s effectiveness.

**Figure 8:**
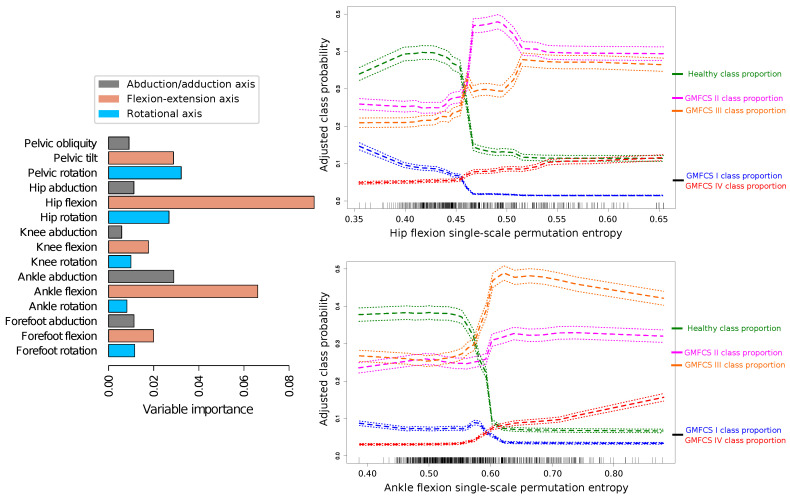
Random forest classification of the patients’ GMFCS stage according to the PE of the joint time series. The left panel shows the importance of the individual features. The higher the value in the X axis of the left panel, the more important the corresponding feature is for an accurate classification. Importance is estimated according to the increase in the classification error when this feature is randomly permuted. The right panels show the adjusted class probability for healthy, GMFCS I, GMFCS II, GMFCS III and GMFCS IV stages according to an RF classification. In the X axis, values of PE of hip flexion (upper plot) and ankle flexion (lower plot) are shown. Different values presented in the split are shown by a small mark in the axis. The left Y axis of the panel indicates the adjusted class probability, while the right one shows the proportion of cycles of the different classes.

We also explored whether single-scale PE could be used to differentiate between cycles from healthy subjects and cycles from patients classified by GMFCS stages. This allows testing the ability of PE to represent the severity of gait impairment in CP; and to further explore which joint movements are mostly related with motor disability. Random forests provide a good concordance (weighted Cohen’s kappa 0.74, 95% confidence interval of 0.69–0.78) between the classification of GMFCS provided by RF and the actual GMFCS stage of the patients. The parameters that were important for the classification (see Figure 8, left panel) were hip flexion PE and ankle flexion PE. Cycles from healthy subjects are characterized by low values of hip flexion (less than 0.45) and ankle flexion (less than 0.55). Cycles from patients with GMFCS I show low PE values for these two movements. This effect seems to be more important in the case of very low hip flexion PE values. Cycles from patients with GMFCS II are featured by increased hip flexion PE, in particular for values between 0.475 and 0.525. Cycles from patients with GMFCS III are particularly characterized by increased PE of ankle flexion (higher than 0.60). Increased values of PE of hip flexion and ankle flexion are associated with higher probability of being classified as a GMFCS IV patient.

## 3. Discussion and Conclusions

This contribution presents the hitherto first kinematic analysis based on permutation entropy, comparing the dynamics of cerebral palsy patients and matched control subjects. This metric, widely used to analyze time series in the biomedical domain and beyond [13], allows the assessment of the complexity of a system not just in terms of the probability distributions associated with its states, but also from a dynamic (i.e., sequence of states) point of view. When applied to multiple gait recordings, representing the dynamics of five different joints in the three-dimensional space, the PE offers a basis for several interesting conclusions.

Kinematic signals of lower limb joint movements during gait cycles display higher permutation entropy in children with CP than in healthy children (see Figure 1). Moreover, we observed that PE is related to the severity of gross motor impairment in patients with CP (Figure 2) and with the severity of kinematic distortion (Figure 5). PE also seems to be related to walking speed, although the magnitude of the change in the PE further depends on the condition of the subject (i.e., control subjects vs. CP patients); see Figure 3 and Figure 4). From the perspective of neural motor control, this fact means that joint movements are less predictable and more complex in children with CP and that this loss of predictability is related to the severity of disability in this disorder.

When the dynamics of each individual joint movement is considered, it can be appreciated that the permutation entropy is modified by the condition of being a CP patient in a non-trivial way: in CP children, the PE increases, but mostly for ankle and forefoot movements. Single-scale PE of pelvic movements is also not linearly correlated with the CP spectrum of performance severity. While PE values decrease at the GMFCS I stage, especially in pelvic movements, they then increase at GMFCS II and beyond; with the exception of pelvic tilt and rotation, for which the permutation entropy values are still lower at GMFCS II. The contribution of the PE of pelvic movements to GMFCS prediction by random forest is small. Pelvic movement PE does not increase with slower walking in healthy subjects, but it does in CP children, and it does not seem to be related to indices of overall kinematic performance; see the lack of significance in Figure 5. Special mention should be made of the case of the pelvic rotation PE, which is negatively correlated with the corresponding MAP score, suggesting that the accuracy of the movement, in terms of supporting the body during gait, depends on the recruitment of a very abnormal kinematic configuration. The dynamics of the hip flexion is strongly modified by the CP condition. The corresponding permutation entropy is decreased at GMFCS I, but increases with higher GMFCS levels. Therefore, hip flexion entropy is an important factor for prediction of GMFCS. The observed permutation entropy is not related to normalized walking speed in healthy subjects, but it increases with slower walking in CP children; see Figure 3. Hip flexion PE is positively correlated with indices of global kinematic abnormality and with their corresponding MAP score. This means that the more erratic the hip flexion movement is, the more impaired is the patient and the more abnormal is the gait. This might suggest an increasing failure of adaptive mechanisms for achieving a hip flexion kinematic configuration efficient enough to provide support and translation as the disease progresses. Non-sagittal hip movement entropy is similar at GMFCS I and in healthy subjects, is slightly increased at GMFCS II and strongly elevated at GMFCS III and GMFCS IV. The hip rotation PE increases with slow walking speed in healthy and CP children and is not related to global indices of gait kinematic performance. In contrast, the hip adduction PE is not related to walking speed in healthy subjects, but it is in CP children, who also have an independent increase in the hip adduction PE due to their condition. The higher the hip adduction PE, the more abnormal is the global gait kinematic performance. Knee movement entropy is mildly decreased at GMFCS I, mildly increased at GMFCS II and very much increased at GMFCS III or IV. Knee flexion PE impacts global kinematic performance, but it is not related to the degree of kinematic abnormality measured by the corresponding MAP score. This suggests that adaptive mechanisms are able to maintain the time series shape of knee flexion (or are unable to change it), but impose very erratic control over it, leading to failures in general gait efficacy. Ankle movement entropy is significantly altered in patients with CP. While the ankle rotation PE seems to be reduced, mainly due to the slower walking speed in CP patients, ankle flexion and abduction PEs are additionally reduced due to CP condition. Ankle abduction PE is negatively related o gait indices, although the effect size is low. Forefoot movement PEs are increased in the CP group, with higher values in the GMFCS III and IV groups. This relationship is stronger in the case of forefoot flexion. The increase in forefoot flexion and rotation PEs is related to the slower walking speed of CP patients. The change of forefoot abduction PE is in contrast higher than might be expected for slower walking speed. The effect on global gait indices is not statistically significant in the case of forefoot flexion and forefoot rotation and slight in the case of forefoot adduction.

Our observations on the relationship of the joint movement PE with kinematic abnormalities point to the conclusion that the former has more impact on the global gait kinematic performance than on the corresponding MAP score of kinematic abnormality of the time series shape. This highlights that the joint movement PE is measuring a domain related to the global control of the neural motor system and management of gait functionality in terms of support and body translation, as opposed to the kinematic configurations recruited for each joint.

The difference of PE between patients with CP and healthy children decreases with lower frequencies as indicated by the analysis of multiscale PE; see Figure 6. From a motor control perspective, motor impairment alters the movement predictability at short intervals, but does not add greater complexity to the global kinematic structure. We hypothesized that abnormal motor control impaired by damage to the corticospinal tracts produces abnormal motor synergies [23] and hyperactive stretch reflexes [24] that lead to an erratic, unpredictable movement at higher frequencies. Why do these abnormalities not lead to a more complex movement at lower frequency? We speculate that if this were to happen, the capability of the motor system to maintain translation capacity would completely disappear.

These results contribute to our understanding of the general principles that regulate the brain motor system’s attempts to compensate the lack of muscle control over the body translation, as produced by the CP lesion. For instance, it seems critical for the system to keep hip and knee dynamics tightly controlled, at the expense of low predictable combinations of pelvis, ankle and feet movements, which necessarily change the biomechanics of the soft tissues. This plastic mechanism can be better achieved in early GMFCS stages, but deteriorates in later stages. Hence, our results suggest and support the idea that hip and knee dynamic rehabilitation strategies should become targets of personalized therapeutic protocols in CP children, to avoid the well-known harmful effects of maladaptations in bone, muscle and soft tissues of the lower extremities.

As any other scientific study, the one proposed here presents some limitations that are worth discussing. An increased size of both groups would augment the precision of the confidence intervals provided by the implemented models, allowing further comparisons between different joint movements. We also believe that the relationships of preferred walking speed with different joint movement PE requires further assessment. Due to the observational nature of our study, we could only establish that, in general, a lower preferred walking speed is associated with higher PE, without a determination of the full causal path for this association. It is important to remind that preferred walking speed is not only an output of the motor system that we were trying to evaluate, but also a biomarker of the motor impairment severity in CP. We used linear mixed models to establish how different is the relationship between walking speed and PE between CP and healthy children, but these statistical approaches are mainly theoretical models that only test associations between variables. To fully understand the relationships between PE and walking speed, we would need to design interventional studies in which the walking speed could be artificially modified. We also believe that future experiments with multimodal measurements (electroencephalography, electromyography and other non-invasive measurement) would help to gain further insights into the biological meaning of PE. Considering all these facts, we believe that PE is an interesting research tool, as it can easily be calculated using the current research protocols in human gait analysis, and it can provide additional information about motor control impairment in human diseases. In this paper, we describe its use as a new methodology to analyze motor control on the basis of kinematic parameters, and as a consequence, it opens new doors towards the future investigation and assessment of the effects of therapeutic interventions on CP gait, or the evaluation of alterations in movement complexity in other neurological diseases.

## 4. Materials and Methods

### 4.1. The Gait Dataset

#### 4.1.1. Participants

A group of 27 healthy school-aged children (5–13 years, Tanner I–II [25]) was used as the control normal sample [26]. This group is our normality reference data sample, used in several other studies. Instrumental gait analysis (IGA) data from 53 school-aged children (5–16 years, Tanner 0–II) with a clinical diagnosis of bilateral spastic CP, recruited in our reference hospitals, were collected. Inclusion criteria were GMFCS I-III or GMFCS IV with GMFCS III in the previous six months, Manual Ability Classification System (MACS) I-II, Communication Function Classification System (CFCS) I, able to walk 7 meters with or without support, no severe cognitive disorder, satisfactory family environment, satisfactory inclusion in the education system, absence of oral anti-spastic treatment or previous injection of botulinum toxin in the previous 6 months and absence of surgical treatment in lower limbs within the previous year. Those two groups of children do not match in number due to the limited availability of volunteers. However, the size of both cohorts has been powerful enough to support the statistical significance of our results. Our local Ethics Committee approved this study, and children were all subjected to examination after parental informed written consent. The work has been carried out in accordance with the Code of Ethics of the World Medical Association (Declaration of Helsinki).

#### 4.1.2. Clinical and 3D-Gait Analysis

Gait analysis was performed with a Codamotion${}^{©}$ system (Charnwood Dynamics Ltd., Rothley, UK). Twenty four light emission markers were attached to the same number of positions of the children’s legs, according to an anthropological segment model designed by the manufacturer, and signals were sampled at 200 Hz while the children were performing the task. Children were incited to walk 10–15 times from one end to the other of a 7 m-long walkway path (5–7 gait cycles per walkway) at their natural, spontaneous speed. They were encouraged to perform the task without support. For those patients who required it, assistance was provided by the parents, who supported the children by holding them by their hands from in front. They were allowed to guide the child and to partially support the child’s weight. The system acquired continuous real-time kinematic data during each complete walk over the walkway. After the acquisition session, individual gait cycles were isolated, by manually marking their beginning (heel contact) and their end (next heel contact of the same foot). Cycles were then reviewed to select those in which the gait was stable, which usually coincided with those obtained from the 3–5 central meters of the walkway. Next, each selected cycle was again reviewed to check the consistency of the signal reception. The whole post-acquisition selection process was performed by two independent reviewers, and additionally with the help of a custom software programmed in R. Such program is designed to detect abnormalities in cycle marking or signal reception and eliminate outliers in discrete kinematic parameters that might mean marker failure or displacement, and which might have escaped the manual revisions. This data validation process resulted in 2–5 valid cycles from each side (left or right leg of the children). For every gait cycle, a matrix of 201 time epochs × 5 joints (pelvis, hip, knee, ankle and forefoot) × sagittal, horizontal and coronal planes was created in the computer interface.

We calculated several indices that are commonly used as indicators of the “quality” of a particular gait pattern. These include the Gait Deviation Index (GDI) [27] and the Gait Profile Score (GPS) [28], which are both approaches used to calculate the distance of one subject’s global gait in relation to a reference dataset, based on 32 kinematic values at key points of the gait cycle. We also calculated the Movement Analysis Profile (MAP) [28], which is a 9-item profile that for each patient expresses the distance of 50 points of 9 kinematic graphs (pelvic tilt, pelvic obliquity, hip flexion/extension, knee flexion/extension and ankle dorsi-plantar flexion) in relation to a reference normal dataset. From this, the Gait Profile Score (GPS) was calculated as an overall index.

IGA and clinical assessments were performed by different researchers. GMFCS stage was registered for CP patients.

### 4.2. Permutation Entropy Analysis

The concept of permutation entropy was initially introduced by Bandt and Pompe to provide researchers with a simple and efficient tool to characterize the complexity of the dynamics of real systems, without the need for introducing arbitrary thresholds or binning procedures: a limitation of existing measures, including entropies, fractal dimensions or Lyapunov exponents [12]. The solution involved focusing on the ordinal structures created by the succession of values in a time series, for the purpose of then calculating the information encoded by such temporal structures.

For the sake of completeness, we here review the main ideas behind permutation entropy, both for single and multiple time scales.

#### 4.2.1. Single-Scale Entropy

The first step in the calculation of the single-scale permutation entropy requires extracting ordinal information from the time series. Given a time series $X=\{x_{t}\}$, with t=1…N, this is divided in maximally overlapping regions of length *D*, such that:

(2) $$s\to (x_{s},x_{s+\tau },\ldots ,x_{s+\tau (D-2)},x_{s+\tau (D-1)}).$$

*D* is called the embedding dimension and controls the quantity of information included in each region, while τ is the embedding delay. *s* further controls the beginning of each region and here assumes all values in the range [1,N−D]. Note that we here fix D=3, due to the limited time series length, and τ=1.

The second step involves associating an ordinal pattern to each region, defined as the permutation $\pi =(r_{0},r_{1},\ldots ,r_{D-1})$ of (0,1,…,D−1), that fulfills:

(3) $$x_{s+r_{0}}\leq x_{s+r_{1}}\leq \ldots \leq x_{s+r_{D-2}}\leq x_{s+r_{D-1}}.$$

In other words, values are sorted in increasing order, with the ordinal pattern corresponding to the required permutation. A simple example may help clarify this concept. Assume a time series X=(1,5,3,4,2); for an embedding dimension of 3, the first region would include the values (1,5,3). In order to sort these three values, the permutation (0,2,1) should be applied (i.e., the first value would not be moved, the third value will be considered next and, finally, the second one): (0,2,1) is then the ordinal pattern associated with the first region of the time series. Similarly, the second region (5,3,4) would be associated with the pattern (1,2,0); and the third region (3,4,2) with (2,0,1).

If all ordinal patterns are expected to appear with the same frequency in a completely random time series, this is seldom true in real-world data. One or several patterns may be under-represented (or completely missing) for several reasons, such as the presence of attractive or forbidden trajectories in the dynamics [21]. The statistical properties of the frequency of appearance of the ordinal patterns can then be used to describe and classify time series generated by different dynamic systems. When the Shannon entropy is used, the result is called the (normalized) permutation entropy:

(4) $$PE=-\frac{1}{\log_{2}D!}\sum_{i=1}^{D!}\pi_{i}\log_{2}\pi_{i}.$$

Note that, in the previous equation, D! represents all the possible permutation patterns and $\pi_{i}$ the frequency of appearance of the *i*-th permutation. Values of PE close to zero indicate time series with a fixed dynamics, i.e., in which only one or a few ordinal patterns can appear, as in monotonously increasing or decreasing sequences. On the other hand, the closer PE is to one, the more random is the time series.

#### 4.2.2. Multi-Scale Entropy

In the previous definition of the permutation entropy, the values used to calculate each permutation where sampled from the time series in a sequential way. This presents the advantage of not discarding any information and thus of including all high-frequency data in the analysis. At the same time, such an approach entails several drawbacks: it is not able to account for the multiple time scales present in the dynamics of physiological systems and is more sensitive to high-frequency noise.

In a way similar to how multi-scale entropies are usually defined [29], we here consider a multi-scale version of the permutation entropy based on analyzing a coarse-grained version of the original time series [30]. Let us suppose that such a time series is given by $X=\{x_{t}\}$, with t=1…N, as before. A new time series *Y* is derived as:

(5) $$y_{t}=\frac{1}{\upsilon }\sum_{i=\upsilon t}^{\upsilon (t+1)}x_{i}.$$

Each value of *Y* thus represents the average of υ values of *X*, drawn sequentially from non-overlapping windows. The permutation entropy is then calculated on *Y*, yielding a result defined as a function of the scale factor υ.

### 4.3. Linear Mixed Models

In order to fully assess the relationship between PE, CP severity and gait speed, we constructed and evaluated three types of linear mixed models.

The first was developed to compare PE values between gait cycles according to the GMFCS level; see Figure 2. PE values were the dependent variable, and a dummy variable of functionality was used to encode GMFCS levels. We adjusted a fixed intercept and four different slopes (beta coefficient) for each GMFCS (I, II, III and IV) and a random intercept for each subject. We calculated the *p*-values for the fixed slope by means of *t*-values’ contrasts and calculated the 95% bias-corrected and accelerated bootstrap interval by using 1000 replicates.

The second model type was designed to assess the effect of CP and normalized walking speed on PE; see Figure 4. The dependent variable was each individual PE value, while fixed slopes (beta coefficients) and intercepts were calculated for normalized walking speed, condition (using healthy as the reference) and the interaction of normalized walking speed with condition; the latter encoded by a binary parameter, 0 representing healthy and 1 CP children. Additionally, a random intercept was introduced for each subject. We calculated the 95% bias-corrected and accelerated bootstrap interval of the three slopes by using 1000 replicates, in order to evaluate how much each independent variable affects the PE. The slope of normalized walking speed indicates how many units of PE increase when normalized walking speed is increased by 1/s. The slope of condition provides information about the average difference in PE between healthy and CP children. The slope of the interaction represents the difference of PE increase when normalized walking speed is increased by 1/s between healthy and CP children; or, in other words, the difference in the effect of walking speed between the two conditions.

The third model type was designed to assess the relationship of gait indices and PE. The dependent variable was either GDI or GPS, while a fixed intercept and fixed slope (beta coefficient) was adjusted for each single-scale PE using a random intercept for each subject. We calculated the 95% bias-corrected and accelerated bootstrap interval of the slope by using 1000 replicates. The slopes represent how much the PE of a particular joint impacts on the general kinematic gait performance in the CP children. Moreover, we have adjusted 9 linear mixed models that used each MAP element as the dependent variable, the corresponding PE as the fixed effect and random intercepts for each subject. In this case, the slopes were used to measure the effect of increasing PE on the kinematic performance of a particular joint movement component.

### 4.4. Classification Task

Random Forests (RFs) are a well-known ensemble learning method for classification, which is based on constructing an ensemble of decision trees and on choosing as output class the one selected by the majority of them [31,32]. RFs are usually recognized as one of the most (if not the most) accurate learning algorithms, showing very little sensitivity to overfitting; an important benefit when dealing with a limited number of instances.

Classification models for CP have been constructed using the random forest algorithm, as implemented in the scikit-learn Python library [33]. Classification models for GMFCS were performed in R by means of the randomForestSRC package [34].

In order to estimate the generalization accuracy of all models, a Leave-One-Out Cross-Validation (LOO CV) strategy has been implemented [35]. It is based on training the classification model *N* separate times (with *N* representing the number of instances), each time on all the data except for one instance, and on calculating the prediction for that point. Finally, the average error and the area under the ROC (AUC) were computed for the RF, which explores differences between healthy and CP children and the weighted Cohen’s kappa in the RF, which classifies between GMFCS stages.

## Figures and Tables

**Table 1:** *p*-values corresponding to two-sided *t*-tests for the null hypothesis that patients and control subjects have identical gait permutation entropy. The ${}^{†}$ symbol denotes those tests that are significant at a $\alpha =6.70\times 10^{-4}$ (i.e., at a significance level of 0.01 with a Šidák correction for multiple testing [20]).

	Abduction-Adduction Axis	Sagittal Axis	Rotational Axis
**Pelvis**	$3.56\times 10^{-11}$ ${}^{†}$	0.183	$5.96\times 10^{-3}$
**Hip**	$4.39\times 10^{-15}$ ${}^{†}$	$4.38\times 10^{-42}$ ${}^{†}$	$9.48\times 10^{-18}$ ${}^{†}$
**Knee**	$3.63\times 10^{-12}$ ${}^{†}$	$4.75\times 10^{-26}$ ${}^{†}$	$9.90\times 10^{-20}$ ${}^{†}$
**Ankle**	$2.13\times 10^{-30}$ ${}^{†}$	$5.34\times 10^{-49}$ ${}^{†}$	$6.33\times 10^{-14}$ ${}^{†}$
**Forefoot**	$4.03\times 10^{-11}$ ${}^{†}$	$1.73\times 10^{-18}$ ${}^{†}$	$4.92\times 10^{-7}$ ${}^{†}$

## Abbreviations

The following abbreviations are used in this manuscript:

CFCS	Communication Function Classification System, scale used to classify the effectiveness of everyday communication of an impaired individual, and specifically of cerebral palsy patients [36].
CP	Cerebral Palsy.
GDI	Gait Deviation Index [27].
GMFCS	Gross Motor Function Classification System, scale describing the impairment of patients based on everyday movements such as sitting and walking [18].
GPS	Gait Profile Score [28].
IGA	Instrumental Gait Analysis.
MACS	Manual Ability Classification System, scale assessing the ability of CP children to handle objects in everyday activities [37].
MAP	Movement Analysis Profile [28].
PE	Permutation Entropy [12,13].
Tanner	Scale of physical development in children and adolescents [25].
